# miR-138-5p Inhibits Vascular Mimicry by Targeting the HIF-1*α*/VEGFA Pathway in Hepatocellular Carcinoma

**DOI:** 10.1155/2022/7318950

**Published:** 2022-05-28

**Authors:** Hongwei Liu, Tao Tang, Xiujin Hu, Weihe Tan, Peng Zhou, Huixian Zhang, Yanmei Liu, Chen Chen, Meng Yang, Meifang Zhou, Shuxia Xuan, Bin Cheng, Weiguo Yin, Jinduan Lin

**Affiliations:** ^1^Department of Laboratory Medicine Center, The Sixth Affiliated Hospital of Guangzhou Medical University, Guangdong Qingyuan, China; ^2^Department of Molecular Diagnostics, Sun Yat-Sen University Cancer Center, Guangdong Guangzhou 510000, China; ^3^Department of Hepatobiliary Surgery, The Sixth Affiliated Hospital of Guangzhou Medical University, Guangdong Qingyuan, China; ^4^Department of Obstetrics and Gynecology, The Sixth Affiliated Hospital of Guangzhou Medical University, Guangdong Qingyuan, China

## Abstract

Tumour vascular mimicry (VM) is the process by which new blood vessels are formed by tumour cells rather than endothelial cells. An increasing number of studies have revealed that the VM process is associated with cancer progression and metastasis. MiR-138-5p has been reported to act as a tumour suppressor in many cancers. However, the role and underlying mechanism of miR-138-5p in hepatocellular carcinoma (HCC) VM remain unclear. In this study, VM density was detected by CD31/periodic acid-Schiff double staining in HCC clinical specimens. We found that miR-138-5p expression correlated strongly and negatively with microvessel density. Additionally, the miR-138-5p mimic or inhibitor decreased or increased, respectively, tube formation capacity in HepG2 and Hep3B cells. Consistent with this finding, miR-138-5p repressed vessel density in vivo. Moreover, miR-138-5p targeted hypoxia-inducible factor 1*α* (HIF-1*α*) and regulated the expression of HIF-1*α* and vascular endothelial growth factor A (VEGFA), which are established classical master regulators for angiogenesis. Consistent with these findings, the HIF-1*α* inhibitor CAY10585 effectively blocked HCC cell VM and VEGFA expression. In conclusion, miR-138-5p inhibits HepG2 and Hep3B cell VM by blocking the HIF-1*α*/VEGFA pathway. Therefore, miR-138-5p may serve as a useful therapeutic target for miRNA-based HCC therapy.

## 1. Introduction

Hepatocellular carcinoma (HCC) is the most common primary liver malignancy and is the third leading cause of cancer-related death worldwide [1, 2]. Accumulated evidence indicates that vascular invasion and metastasis play important roles in the high mortality rate of patients with HCC [3, 4]. Tumour growth and metastasis require an adequate blood supply to transport nutrients and oxygen and remove metabolic waste, providing a pathway for tumour metastasis and stimulating growth of the tumour mass. Along with endothelial vessels (EVs), tumour vascular mimicry (VM) occurs via the generation of a de novo vascular network by tumour cells to supply nutrients for sustaining tumour growth. EVs and VM are both important processes in the tumorigenicity and metastasis of HCC.

MicroRNAs (miRNAs) are noncoding RNAs approximately 22 nucleotides in length that have an important function in many essential physiological and pathological processes. miRNAs regulate target genes at the posttranscriptional level by binding to their 3′-UTR [5, 6]. It has been reported that miR-138-5p acts as a tumour suppressor to inhibit HCC growth, metastasis, glycolysis, and oxaliplatin resistance in vivo and in vitro [7–9]. Moreover, miR-138-5p inhibits HCC progression by inhibiting hepatitis B virus replication and expression. However, the roles of miR-138-5p in HCC VM remain largely unknown.

Recent reports have demonstrated that miR-138-5p inhibits endothelial progenitor cell proliferation by inhibiting hypoxia-inducible factor 1*α* (HIF-1*α*) [10, 11]. HIF-1*α* is an important molecule for EVs and VM. Given that miR-138-5p affects both tumour cells and endothelial cells and regulates the expression level of HIF-1*α* in endothelial cells, we hypothesized that miR-138-5p might suppress tumour growth by blocking blood nutrient supply. The effects of miR-138-5p on VM have not been reported in any type of cancer. In this study, we found that miR-138-5p inhibited VM and regulated HIF-1*α*/vascular endothelial growth factor A (VEGFA) expression by targeting HIF-1*α* in HCC cells.

## 2. Materials and Methods

### 2.1. Tissue Specimens

Fresh human HCC and corresponding paratumour tissues were obtained from surgical specimens immediately after their resection from patients who underwent routine surgery at Qingyuan Hospital, The Six Affiliated Hospital of Guangzhou Medical University (Qingyuan, China). Patients who received preoperative irradiation, chemotherapy, immunotherapy, or targeted therapy were excluded. All patients were confirmed to have HCC by postoperative pathology. Finally, 23 pairs of fresh human HCC and corresponding paratumour tissues were included in the study. The research protocol was approved by the Ethics Committee of The Six Affiliated Hospital of Guangzhou Medical University (No. QPH-IRB-A0153). All experiments were carried out in accordance with the international ethical guidelines for biomedical research involving human subjects.

### 2.2. Antibodies and Inhibitor

Antibodies against CD31 (Abcam, ab182981, Cambridge, UK), HIF-1*α* (Abcam, ab51608, Cambridge, UK), and VEGFA (ABclonal, A12303, Wuhan, China) were used for western blotting and immunohistochemistry (IHC). CAY10585 (Abcam, ab144422, Cambridge, UK) was used to inhibit HIF-1*α*.

### 2.3. Cell Culture

Human HCC cell lines, including HepG2 (hepatoblastoma cell line), Hep3B, MHCC97H, MHCC97L, Huh7, SK-Hep1, and MIHA, were purchased from the Chinese Academy of Sciences. MIHA human immortalized normal hepatocytes were used as normal control cells. The cells were cultured in Dulbecco's modified Eagle's medium (DMEM, Gibco, Bengjing, China) supplemented with 10% foetal bovine serum (Corning, Australia) and 1% penicillin–streptomycin (Gibco, Gaithersburg, MD, USA) and incubated at 37°C in a humidified atmosphere containing 5% CO_2_. Under hypoxic conditions, cells were cultured in an atmosphere of 1% O2.

### 2.4. RNA Extraction and Quantitative Real-Time Polymerase Chain Reaction (qRT–PCR)

Total RNA was extracted from cultured cells or human tissues by using TRIzol reagent (Takara, Dalian, China). qRT–PCR assays were conducted to determine the mRNA levels by using a SYBR Green PCR Master Mix kit (Takara, Dalian, China). *β*-Actin was used as an internal control to analyse HIF-1*α* and VEGFA mRNA levels. U6 expression was used as an internal control for analysing miR-138-5p levels in HCC tissue and HCC cell lines. The 2^-*ΔΔ*CT^ method was used to assess the relative levels of HIF-1*α* and VEGFA mRNA levels. All assays were evaluated in at least three repeats, and all the results are shown as the mean ± standard deviation (SD) for analysis. The primers are shown in Supplemental Table 1.The median expression level was used as the cut-off. Low expression was classified as values below the 50th percentile. High miR-138-5p expression was classified as values at or above the 50th percentile.

### 2.5. RNA Interference and Transfection

Hep3B and HepG2 cells were seeded into a 6-well plate at 30-50% confluence. Twenty-four hours later, the cells were transfected with the HIF-1*α* inhibitor CAY10585 or transfected with a miR-138-5p mimic (Sangon Biotech, Shanghai, China) or a miR-138-5p inhibitor (Sangon Biotech, Shanghai, China) using Lipofectamine 2000 reagent (Invitrogen, Carlsbad, USA) according to the manufacturer's instructions. Cells transfected with a scramble control siRNA (NC) were used as controls. The cells were harvested 48 h after transfection. The sequences of the siRNA against HIF-1*α*, miR-138-5p mimic, and miR-138-5p inhibitor are listed in Supplemental Table 1.

### 2.6. Luciferase Assay

To test the direct binding of miR-138-5p to the target gene HIF-1*α*, a luciferase reporter assay was performed as previously described. The fragment of HIF-1*α* 3′UTR containing miR-138-5p binding sites or mutated sites was inserted into the pmirGLO vector (Promega, Madison, USA) to generate HIF-1*α* 3′UTR-WT or HIF-1*α*3′UTR-MUT. Then, the constructed plasmids were transfected into HepG2 cells together with miR-NC or miR-138-5p. Luciferase activity was measured 48 h posttransfection using a dual-luciferase assay kit (Promega, Madison, USA).

### 2.7. Immunohistochemistry

We performed IHC assays by using an IHC kit (Boster Bio-Engineering Company, Wuhan, China) to detect the protein levels of VEGFA and HIF-1*α*. Tumour tissue was embedded in paraffin and cut into 4 *μ*m sections. Sections were deparaffinized with xylene and rehydrated with graded ethanol then immersed in citrate and boiled at 126°C for 1-2 min to recover antigen. Endogenous peroxidase was blocked with H_2_O_2_ (3%) for 20 min. Each section was first incubated with 50 *μ*L of goat serum (16210064, Thermo Fisher, Waltham, MA, USA) for 15 min at room temperature, followed by 50 *μ*L of primary antibody against VEGFA (1 : 200) or HIF-1*α* (1 : 200) in a humidified chamber at 4°C. After removing the primary antibody, each section was incubated with 50 *μ*L of the secondary antibody goat anti-rabbit IgG H&L (HRP) (ab205718, 1 : 2000, Abcam, UK). Positively stained cells were stained with 1 mL of 0.05% DAB solution (11718096001, Sigma–Aldrich, USA) followed by haematoxylin (H9627, Sigma–Aldrich, USA) for 3 min. We also used CD31/periodic acid-Schiff (PAS) double staining to detect VM density. The staining intensity was represented by scores as follows: 0, negative; 1, weak; 2, medium; and 3, strong. The staining extent scores were as follows: 0 represents <10%, 1 represents 11-25%, 2 represents 26-50%, 3 represents 51-75%, and 4 represents 76-100%. The final protein expression score was calculated as the intensity score × extent score and ranged from 0 to 12.

### 2.8. Protein Extraction and Western Blotting

Protein extraction kits (Keygen Biotech, Nanjing, China) were used to extract protein from HepG2 and Hep3B cells. The protein concentrations were determined by a bicinchoninic acid protein assay kit (Keygen Biotech, Nanjing, China). WB was conducted according to the literature. Proteins were separated by 8–12% sodium dodecyl sulfate–polyacrylamide gel electrophoresis (SDS–PAGE) and transferred to a polyvinylidene difluoride (PVDF) membrane. Membranes were incubated with antibodies against VEGFA (1 : 1000) or HIF-1*α* (1 : 1000) and horseradish peroxidase- (HRP-) conjugated secondary antibodies (1 : 10000). Quantification was performed using ImageJ software.

### 2.9. Bioinformatics Analysis

TargetScan 3.1 online software (https://www.targetscan.org/) was used to predict the putative target genes of miR-138-5p. The species was “human,” and “miR-138-5p” was entered as the miRNA name.

### 2.10. Tube Formation Assay

We chose the HepG2 and Hep3B cell lines to carry outgain- and loss-of-function experiments according to the basic expression level of miR-138-5p and the basic tube formation ability. We used Matrigel (BD Biosciences, San Jose, USA) to perform the tube formation assay. first, we add 50 ul Matrigel ploymerization into a 96-well plate, keeping in 37 degrees for 30 minutes, and then put the HepG2 and Hep3B cell treated with miR-138-5p mimic and inhibitor in to the 96-well plate. After 6 h of incubation (37°C, 5% CO_2_), tube numbers were counted under an IX71 inverted microscope.

### 2.11. Xenograft Tumours In Vivo

All experimental procedures involving animals were performed in accordance with the “Guide for the Care and Use of Laboratory Animals” prepared by the National Academy of Sciences and published by the National Institutes of Health (NIH publication 86–23 revised 1985). Our animal investigations were performed in accordance with the institutional guidelines and were approved by the Animal Experimental Committee of The Sixth Affiliated Hospital of Guangzhou Medical University. Four-week-old male BALB/c nude mice were purchased from the Guangdong Experimental Animal Center of the Chinese Academy of Sciences and were bred and maintained in specific pathogen-free conditions. All mice were housed in a pathogen-free barrier facility with food and water ad libitum. Cells from each group were resuspended in serum-free DMEM at a density of 5 × 10^7^ cells per mL, and then, 0.1 mL of the suspension was injected into the backs of the nude mice. At the end of the treatment cycle (4 weeks later), all mice were euthanized by CO_2_ asphyxiation. Tumour tissues were obtained for CD31/periodic acid-Schiff (PAS) double staining.

### 2.12. Survival Analysis

We performed Kaplan-Meir survival analysis on “The Cancer Genome Atlas (TCGA)” publicly available datasets using the ‘Kaplan-Meir Plotter' database (https://kmplot.com/analysis/). The miR-138-5p data were based on the miRNA subset. The HIF-1*α* and VEGFA mRNA cohorts were based on the RNA-seq subset. We also split the dataset by autoselecting the best cut-off in the system.

### 2.13. Statistical Analysis

Data were shown as *χ* ± SD, and all data were obtained from at least three replicates. Data were analysed by SPSS 13.0 (SPSS, Inc.) and GraphPad Prism (GraphPad Software, Inc.). The chi-square test was used to examine the difference in miR-138-5p expression between paratumour and tumour tissues. Student's *ttest* or one-way ANOVA was used to assess the differences among groups. Statistical significance was set as follows: ^∗^represents *P* < 0.05, ^∗∗^represents *P* < 0.01, and ^∗∗∗^represents *P* < 0.001.

## 3. Results

### 3.1. miR-138-5p Was Negatively Associated with VM Density and Poor Prognosis in HCC Patients

**Figure 1:**
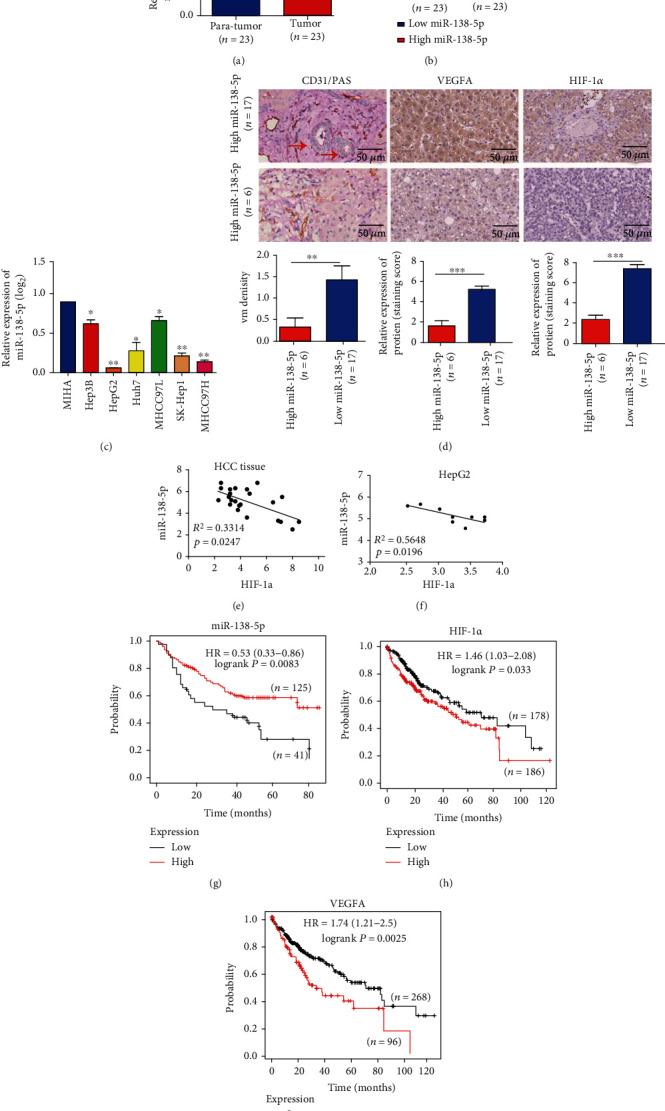
miR-138-5p is frequently downregulated in HCC tissues and HCC cell lines compared to control, and low miR-138-5p expression is associated with a high VM density and high HIF-1*α* and VEGFA levels and indicates a poor prognosis in HCC patients. (a) and (b) RT–PCR analysis showed that miR-138-5p was more highly expressed in HCC tissues than in paratumour tissues. (c) RT–PCR analysis showed that miR-138-5p was expressed at lower levels in HCC cell lines than in human normal hepatocytes (MIHA cells).(d) CD31/PAS double staining was used to assess VM formation (red arrow). Immunohistochemical staining was used to assess HIF-1*α* and VEGFA expression. The results showed that miR-138-5p expression was related to VM density and HIF-1*α* and VEGFA levels. (e) The correlation between miR-138-5p and HIF-1*α* mRNA levels in 23 HCC tissues. The *Δ*Ct values were subjected to Pearson correlation analysis. (f) The correlation between miR-138-5p and HIF-1*α* mRNA levels in the HepG2 cell line (9 repeats). The *Δ*Ct values were subjected to Pearson correlation analysis. (g)–(i) The log-rank (Mantel–Cox) test showed that HCC patients with low miR-138-5p levels and high HIF-1*α* and VEGFA levels demonstrated worse OS than other patients from the KM-Plotter database. Statistical significance was set as follows: ^∗^represents *P* < 0.05, ^∗∗^represents *P* < 0.01, and ^∗∗∗^represents *P* < 0.001.

To determine the relationship between miR-138-5p expression and HCC, we conducted qRT–PCR to examine miR-138-5p expression levels. The median expression level was used as the cut-off. Low expression of miR-138-5p in 21 tissues (4 paratumour and 17 tumour tissues) was classified as values below the 50th percentile. High miR-138-5p expression in 25 tissues (19 paratumour and 6 tumour tissue) was classified as values at or above the 50th percentile. As shown in Figures 1(a) and 1(b), the expression level of miR-138-5p in HCC tissues was lower than that in paratumour tissues. Similarly, miR-138-5p was expressed at lower levels in HCC cell lines than in the human normal hepatocyte cell line (MIHA) (Figure 1(c)). Furthermore, we found that low expression of miR-138-5p was associated with a high VM density and high HIF-1*α* and VEGFA levels (Figure 1(d)), and the expression of miR-138-5p was negatively correlated with HIF-1*α* mRNA levels (Figures 1(e) and 1(f)). Furthermore, the survival results from the Online Kaplan–Meier Plotter database indicated that low miR-138-5p and high HIF-1*α* and VEGFA mRNA levels were associated with a poor prognosis in HCC patients (Figures 1(g)–1(i)). Together, these data suggested that downregulation of miR-138-5p was associated with a high VM density and high HIF-1*α* and VEGFA levels and might be associated with HCC progression.

### 3.2. miR-138-5p Repressed VM Formation in HCC Cells

**Figure 2:**
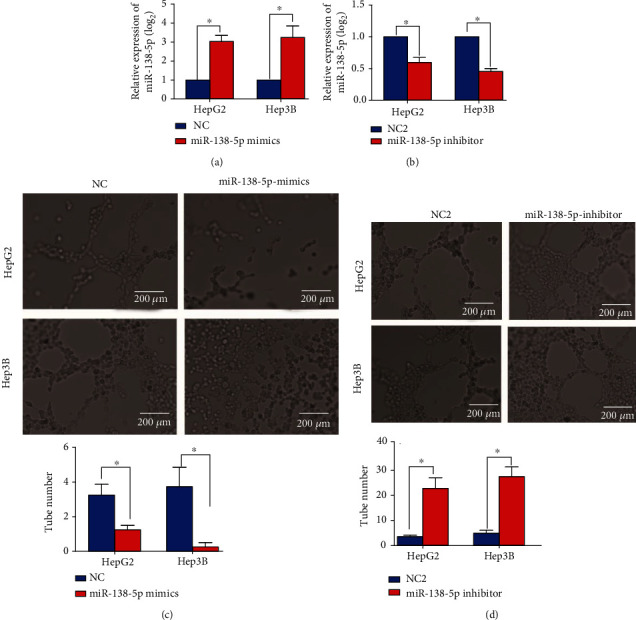
miR-138-5p reduces HepG2/Hep3B tumour cell tube formation. (a) and (b) Transfection efficiencies of the miR-138-5p mimic (A) and miR-138-5p inhibitor (B) in HCC cells. (c) Compared with the control, the miR-138-5p mimic reduced HepG2/Hep3B tumour cell tube formation. (d) The miR-138-5p inhibitor enhanced HepG2/Hep3B tumour cell tube formation. All experiments were repeated 3 times. Data are shown as the mean ± SD. ^∗^Represents *P* < 0.05.

Our data show that the lower level of miR-138-5p in HCC tissues indicates a possible high VM density and poor prognosis. We sought to determine whether miR-138-5p participates in the process of HCC VM. To this end, we conducted a tube formation assay to assess the effects of miR-138-5p knockdown/overexpression by a miR-138-5p inhibitor or miR-138-5p mimic. The expression levels of miR-138-5p after cell transfection with the miR-138-5p mimic or inhibitor are shown in Figures 2(a) and 2(b). Compared with the control group, HCC cells transfected with the miR-138-5p inhibitor exhibited a significantly enhanced VM formation ability, whereas this process was significantly repressed in HCC cells transfected with the miR-138-5p mimic (Figures 2(c) and 2(d)). Our results suggested that the dysregulation of miR-138-5p expression in HCC cells might play an important role in HCC VM in vitro.

### 3.3. miR-138-5p Repressed Vessel Density In Vivo

**Figure 3:**
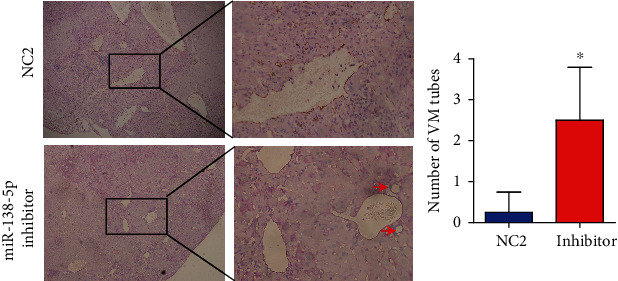
miR-138-5p repressed vessel density in vivo. Nude mouse results showed that when injected with HCC cells treated with miR-138-5p inhibitor, the density of VM was increased. The red arrow shows the CD31(-)/PAS(+) VM structures. All experiments were repeated 5 times. Data are shown as the mean ± SD. ^∗^Represents *P* < 0.05.

To determine the effects of miR-138-5p on HCC VM formation, we injected HepG2 cells that were treated with miR-138-5p inhibitor into the backs of nude mice. Mice injected with cells treated with miR-138-5p inhibitor had a higher VM density in the tumour tissue than the control group (Figure 3).

### 3.4. miR-138-5p Repressed the VM Capacity by Directly Targeting HIF-1*α* in HCC Cells

**Figure 4:**
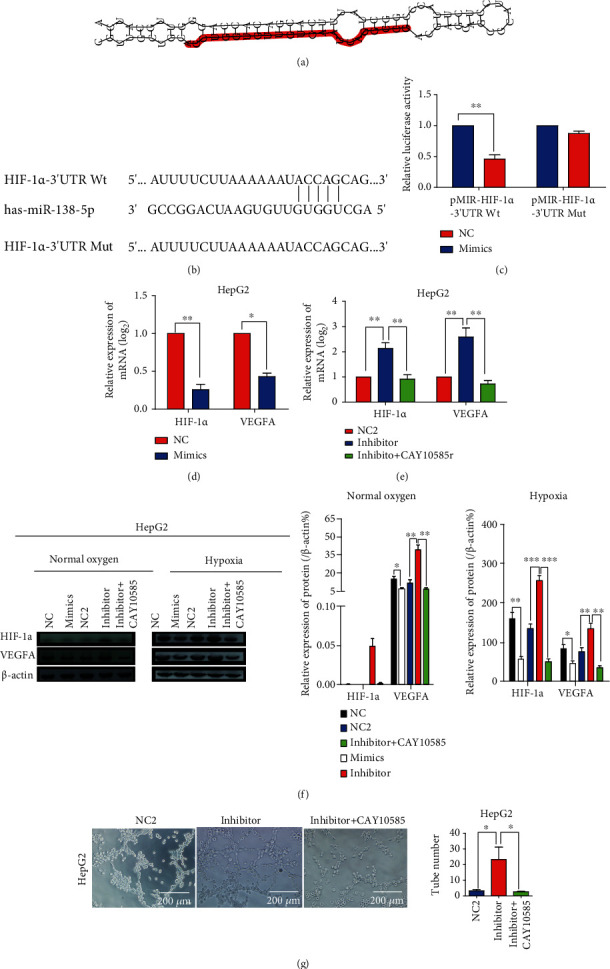
miR-138-5p reduces tumour cell tube formation by targeting HIF-1*α*/VEGFA signalling. (a) The structure of miR-138-5p was predicted from miRTarBase (http://mirtarbase. http://cuhk.edu.cn/). (b) The predicted binding site of miR-138-5p in the 3′-UTR of HIF-1*α* and the mutated 3′-UTR of HIF-1*α*. (c) A luciferase reporter plasmid containing the wild-type HIF-1*α* 3′-UTR or the mutant HIF-1*α* 3′-UTR was transfected into 293 T cells alone or cotransfected with the NC or miR-138-5p mimic, and luciferase activity was measured. (d) and (e) qRT–PCR detection of HIF-1*α* and VEGFA mRNA expression in HCC cells treated with the miR-138-5p mimic (d) or inhibitor (e). (f) Western blot detection of HIF-1*α* and VEGFA protein expression in HCC cells transfected with the miR-138-5p mimic or inhibitor or with the HIF-1*α* inhibitor CAY10585 under normal oxygen and hypoxic conditions. (g) The HIF-1*α* inhibitor CAY10585 reversed the effects of the miR-138-5p inhibitor on tube formation by HCC cells. Data are shown as the mean ± SD. All experiments were repeated at least 3 times. ^∗^Represents *P* < 0.05; ^∗∗^Represents *P* < 0.01; ^∗∗∗^Represents *P* < 0.001.

HIF-1*α* is associated with VM [4, 12]. Therefore, the effect of miR-138-5p downregulation on HIF-1*α* expression was investigated. The predicted structure of miR-138-5p from miRBase (https://mirtarbase.cuhk.edu.cn/) is shown in Figure 4(a). TargetScan 3.1 online software predicted that HIF-1*α* was one of the putative target genes of miR-138-5p. The predicted results of the binding site of miR-138-5p with HIF-1*α* are shown in Figure 4(b). A luciferase reporter assay showed that HIF-1*α* was a direct target of miR-138-5p (Figure 4(c)). As expected, transfection with the miR-138-5p mimic significantly reduced HIF-1*α* and VEGFA mRNA and protein expression in HCC cells (Figures 4(d) and 4(f)). Transfection with the miR-138-5p inhibitor significantly enhanced HIF-1*α* and VEGFA mRNA and protein expression in HCC cells, and the HIF-1*α* inhibitor reversed the effects of the miR-138-5p inhibitor on HIF-1*α* and VEGFA mRNA and protein expression (Figures 4(e) and 4(f)). The HIF-1*α* inhibitor CAY10585 also reversed the effects of the miR-138-5p inhibitor on HCC cell tube formation (Figure 4(g)). Therefore, we inferred that miR-138-5p influenced VM, at least in part, by regulating HIF-1*α* and VEGFA in HCC cells.

## 4. Discussion

Angiogenesis is an important hallmark of tumours [13]. Tumours need an adequate blood supply to ensure enough nutrition for further development. In addition to secreting angiogenic substances to induce endothelial cells to form tubes to produce more blood vessels [14], tumour cells can form new tube structures to increase blood supply, which is called VM. VM is a recently described mechanism in which a blood supply is provided by tumour cells rather than endothelial cells, and it has been observed in certain highly aggressive tumours. miRNAs, small noncoding RNAs of approximately 22 nucleotides in length, have been suggested to be important modulatory factors in the process of VM in HCC [15].

It has been reported that a lower miR-138-5p level is associated with tumour progression and metastasis and that miR-138-5p acts as a tumour suppressor in most cancers [16–19]. In HCC research, recent studies have shown that miR-138-5p is frequently downregulated in HCC compared to controls and inhibits the occurrence and development of HCC. For example, Liu et al. [20] indicated that miR-138-5p could target and inhibit SOX9 expression and thus repress cell proliferation and invasion in HCC. Lin et al. [21] found that miR-138-5p suppresses metastasis and tumorigenesis by enhancing vimentin expression and ubiquitination of cyclin E in HCC. However, the role of miR-138-5p in HCC VM has not been determined.

This study represents the first investigation of miR-138-5p in relation to VM in HCC. In this study, we found that miR-138-5p was downregulated in HCC tissues, and low miR-138-5p expression was significantly correlated with a poor prognosis of patients. Moreover, low miR-138-5p expression was related to a high EV density. Given the different basic tube formation abilities in different cell lines, we chose the HepG2 and Hep3B cell lines to carry outgain- or loss-of-function experiments according to the basic expression level of miR-138-5p and the basic tube formation ability. Consistently, in vitro experiments confirmed that miR-138-5p functioned as a tumour suppressor gene by inhibiting HCC VM.

miRNAs exert biological functions by regulating target genes via binding to their 3′-UTR. We found that HIF-1*α* might be a target gene of miR-138-5p by using bioinformatics tools. By conducting a dual-luciferase reporter assay, we demonstrated that miR-138-5p directly binds to the 3′-UTR of HIF-1*α* mRNA and that miR-138-5p expression is negatively related to HIF-1*α* mRNA expression in HCC cell lines. HIF-1*α* is a hypoxia-responsive factor that responds to hypoxia by activating the master regulator of the transcription of many genes and participates in cell energy metabolism, angiogenesis, proliferation, and apoptosis. HIF-1*α* is frequently upregulated in many tumours. Studies have shown that HIF-1*α* acts as an oncogene and participates in tumour growth, metastasis [22], metabolic rewiring, and chemoresistance [23, 24]. In addition, HIF-1*α* promotes tumour angiogenesis and VM and may be a prognostic biomarker for some tumours [25–27]. Similarly, HIF-1*α* promotes cell proliferation and migration in HCC [28]. In this study, we found that the miR-138-5p inhibitor enhanced HCC cell tube formation. Additionally, miR-138-5p targeted HIF-1*α* and downregulated its expression, and the effects of the miR-138-5p inhibitor on HCC cell tube formation were reversed by the HIF-1*α* inhibitor CAY10585. All the results indicated that miR-138-5p suppressed VM in HCC by targeting HIF-1*α* and that HIF-1*α* was a medium for miR-138-5p in the process of HCC VM. Consistent with our results, Wang et al., Zhang et al., and Pinyol et al. found that HIF-1*α* played an important role in promoting HCC VM [4, 29, 30].

Increasing evidence demonstrates that VEGFA is an important downstream factor of HIF-1*α*. In this study, we found that when cells were transfected with the miR-138-5p mimic, HIF-1*α* and VEGFA expression was significantly reduced. Accumulating evidence has shown that VEGFA plays a key role in tumour angiogenesis and VM [12, 31]. Therefore, miR-138-5p partly represses HCC VM by downregulating the expression of VEGFA. Considering the above findings, we demonstrated that miR-138-5p might act as a tumour suppressor by inhibiting VM by targeting HIF-1*α* and downregulating VEGFA and HIF-1*α* expression.

In conclusion, our studies showed that miR-138-5p was frequently downregulated in HCC tissues compared to paratumour tissues. Lower miR-138-5p expression was related to a high VM density and high VEGFA and HIF-1*α* levels in HCC tissues. In addition, low miR-138-5p expression indicated a poor prognosis in HCC patients. In terms of the mechanism, miR-138-5p targeted HIF-1*α*, which is one of the upstream regulators of VEGFA, and HIF-1*α* and VEGFA participated in HCC VM. Thus, the current study links miR-138-5p, VEGFA, and HIF-1*α* to VM in HCC, and the role of miR-138-5p in HCC VM is clearly indicated. MiR-138-5p may act as a potential therapeutic target in HCC.

## Data Availability

We declare that all data generated or analysed during this study are included in this published article and its supplementary information files.

## Abbreviations

VEGFA: Vascular endothelial growth factor A
qRT–PCR: Quantitative real-time polymerase chain reaction
HCC: Hepatocellular carcinoma
VM: Vascular mimicry
OS: Overall survival
IHC: Immunohistochemistry
HIF-1*α*: Hypoxia-inducible factor 1*α*.

## Supplementary Materials

Supplementary Materials Table 1: sequences of the primers used in this study.

## References

[1] Villanueva A. (2019). Hepatocellular carcinoma. *The New England Journal of Medicine*.

[2] Bray F., Ferlay J., Soerjomataram I., Siegel R. L., Torre L. A., Jemal A. (2018). Global cancer statistics 2018: GLOBOCAN estimates of incidence and mortality worldwide for 36 cancers in 185 countries. *CA: a Cancer Journal for Clinicians*.

[3] Xu J., Zhang Y., Wang Y. (2018). Correlation of KAI1, CD133 and vasculogenic mimicry with the prediction of metastasis and prognosis in hepatocellular carcinoma. *International Journal of Clinical and Experimental Pathology*.

[4] Wang M., Zhao X., Zhu D. (2017). HIF-1*α* promoted vasculogenic mimicry formation in hepatocellular carcinoma through LOXL2 up-regulation in hypoxic tumor microenvironment. *Journal of Experimental & Clinical Cancer Research*.

[5] Kanchan R. K., Siddiqui J. A., Mahapatra S., Batra S. K., Nasser M. W. (2020). microRNAs orchestrate pathophysiology of breast cancer brain metastasis: advances in therapy. *Molecular Cancer*.

[6] Yang H., Ma Q., Wang Y., Tang Z. (2020). Clinical application of exosomes and circulating microRNAs in the diagnosis of pregnancy complications and foetal abnormalities. *Journal of Translational Medicine*.

[7] Li B., Zhao H., Song J., Wang F., Chen M. (2020). LINC00174 down-regulation decreases chemoresistance to temozolomide in human glioma cells by regulating miR-138-5p/SOX9 axis. *Human Cell*.

[8] Wang Z., Yao Y. J., Zheng F. (2017). mir-138-5p acts as a tumor suppressor by targeting pyruvate dehydrogenase kinase1 in human retinoblastoma. *European Review for Medical and Pharmacological Sciences*.

[9] Wang X., Zhao Y., Cao W. (2017). miR-138-5p acts as a tumor suppressor by targeting hTERT in human colorectal cancer. *International Journal of Clinical and Experimental Pathology*.

[10] Zhang Y., Du X., Li W. (2018). Resveratrol improves endothelial progenitor cell function through miR-138 by targeting focal adhesion kinase (FAK) and promotes thrombus resolution in vivo. *Medical Science Monitor*.

[11] Zhou W., Zhou W., Zeng Q., Xiong J. (2017). MicroRNA-138 inhibits hypoxia-induced proliferation of endothelial progenitor cells via inhibition of HIF-1*α*-mediated MAPK and AKT signaling. *Experimental and Therapeutic Medicine*.

[12] Wang H. F., Wang S., Zheng M. (2019). Hypoxia promotes vasculogenic mimicry formation by vascular endothelial growth factor a mediating epithelial-mesenchymal transition in salivary adenoid cystic carcinoma. *Cell Proliferation*.

[13] Hanahan D., Weinberg R. A. (2011). Hallmarks of cancer: the next generation. *Cell*.

[14] Khorshidi A., Dhaliwal P., Yang B. (2016). Noncoding RNAs in tumor angiogenesis. *Advances in Experimental Medicine and Biology*.

[15] Yang J., Lu Y., Lin Y. (2016). Vascular mimicry formation is promoted by paracrine TGF-*β* and SDF1 of cancer- associated fibroblasts and inhibited by miR-101 in hepatocellular carcinoma. *Cancer Letters*.

[16] Song N., Li P., Song P. (2020). MicroRNA-138-5p suppresses non-small cell lung cancer cells by targeting PD-L1/PD-1 to regulate tumor microenvironment. *Frontiers in Cell and Development Biology*.

[17] Shi T., Li R., Zhao L. (2020). Long noncoding RNA UCA1 regulates CCR7 expression to promote tongue squamous cell carcinoma progression by sponging miR-138-5p. *Neoplasma*.

[18] Milanesi E., Dobre M., Bucuroiu A. I. (2020). miRNAs-based molecular signature for KRAS mutated and wild type colorectal cancer: an explorative study. *Journal of Immunology Research*.

[19] Zhang D., Liu X., Zhang Q., Chen X. (2020). miR-138-5p inhibits the malignant progression of prostate cancer by targeting FOXC1. *Cancer Cell International*.

[20] Liu Y., Zhang W., Liu K., Liu S., Ji B., Wang Y. (2016). miR-138 suppresses cell proliferation and invasion by inhibiting SOX9 in hepatocellular carcinoma. *American Journal of Translational Research*.

[21] Lin S. L., Lin Y. H., Chi H. C. (2020). A novel long non-coding RNA-01488 suppressed metastasis and tumorigenesis by inducing miRNAs that reduce vimentin expression and ubiquitination of cyclin E. *Cell*.

[22] Zhao J., Xiao A., Liu C. (2020). The HIF-1A/miR-17-5p/PDCD4 axis contributes to the tumor growth and metastasis of gastric cancer. *Signal Transduction and Targeted Therapy*.

[23] Khan H., Anshu A., Prasad A. (2019). Metabolic rewiring in response to Biguanides is mediated by mROS/HIF-1a in malignant lymphocytes. *Cell Reports*.

[24] Tang J. H., Ma Z. X., Huang G. H. (2016). Downregulation of *HIF-1a* sensitizes U251 glioma cells to the temozolomide (TMZ) treatment. *Experimental Cell Research*.

[25] Li W., Zong S., Shi Q., Li H. J., Xu J., Hou F. (2016). Hypoxia-induced vasculogenic mimicry formation in human colorectal cancer cells: involvement of HIF-1a, claudin-4, and E-cadherin and vimentin. *Scientific Reports*.

[26] Cai F., Xu C., Pan X. (2016). Prognostic value of plasma levels of HIF-1a and PGC-1a in breast cancer. *Oncotarget*.

[27] Cheng Z., Fu J., Liu G., Zhang L., Xu Q., Wang S. Y. (2018). Angiogenesis in JAK2 V617F positive myeloproliferative neoplasms and ruxolitinib decrease VEGF, HIF-1 enesis in JAK2 V617F positive cells. *Leukemia & Lymphoma*.

[28] Chen Y., Huang F., Deng L. (2019). HIF-1-miR-219-SMC4 regulatory pathway promoting proliferation and migration of HCC under hypoxic condition. *BioMed Research International*.

[29] Zhang J. G., Zhou H. M., Zhang X. (2020). Hypoxic induction of vasculogenic mimicry in hepatocellular carcinoma: role of HIF-1 *α*, RhoA/ROCK and Rac1/PAK signaling. *BMC Cancer*.

[30] Pinyol R., Montal R., Bassaganyas L. (2019). Molecular predictors of prevention of recurrence in HCC with sorafenib as adjuvant treatment and prognostic factors in the phase 3 STORM trial. *Gut*.

[31] Liang H., Xiao J., Zhou Z. (2018). Hypoxia induces miR-153 through the IRE1*α*-XBP1 pathway to fine tune the HIF1*α*/VEGFA axis in breast cancer angiogenesis. *Oncogene*.

[32] Hongwei L., Xiujin H., Weihe T. (2021). miR-138-5p inhibits vascular mimicry by targeting the HIF-1*α*/VEGFA pathway in hepatocellular carcinoma. https://www.researchsquare.com/article/rs-1080163/v1.

