# Supplementary material for: miR-138-5p Inhibits Vascular Mimicry by Targeting the HIF-1α/VEGFA Pathway in Hepatocellular Carcinoma
Source: J Immunol Res. 2022 May 28;2022:7318950. doi: 10.1155/2022/7318950 (PMC9167126; doi:10.1155/2022/7318950)
Supplement: Supplementary Materials — Table 1: sequences of the primers used in this study. [file 7318950.f1.pdf]

Supplement table 1 Sequences of primers sequences used in this study

Name		Sequences
miR-138-5p	sense	GCTTAAGGCACGCGG
	antisense	GTGCAGGGTCCGAGG
U6	sense	CTCGCTTCGGCAGCACA
	antisense	AACGCTTCACGAATTTGCGT
β-actin	sense	TGGCACCCAGCACAATGAA
	antisense	CTAAGTCATAGTCCGCCTAGAAGCA
HIF-1α	sense	GAACGTCGAAAAGAAAAGTCTCG
	antisense	CCTTATCAAGATGCGAACTCACA
VEGFA	sense	AGGGCAGAATCATCACGAAGT
	antisense	AGGGTCTCGATTGGATGGCA
miR-138-5p	mimics	AGCUGGUGUUGUGAAUCAGGCCG
	mimics-NC	UUCUCCGAACGUGUCACGUTT
	inhibitor	CGGCCUGAUUCACAACACCAGCU
	inhibitor-NC	CAGUACUUUUGUGUAGUACAA
